# Biomarkers of acute respiratory distress syndrome in adults hospitalised for severe SARS-CoV-2 infection in Tenerife Island, Spain

**DOI:** 10.1186/s13104-020-05402-w

**Published:** 2020-12-09

**Authors:** Juan Marco Figueira Gonçalves, José María Hernández Pérez, Marco Acosta Sorensen, Aurelio Luis Wangüemert Pérez, Elena Martín Ruiz de la Rosa, José Luis Trujillo Castilla, David Díaz Pérez, Yolanda Ramallo-Fariña

**Affiliations:** 1 Pneumology and Thoracic Surgery Service, University Hospital Nuestra Señora de Candelaria, Santa Cruz de Tenerife, Spain; 2 Pneumology Service, San Juan de Dios Hospital, Tenerife, Spain; 3 Foundation of the Canary Islands Health Research Institute (FIISC), Santa Cruz de Tenerife, Spain; 4 Health Services Research On Chronic Patients Network (REDISSEC), Madrid, Spain

**Keywords:** COVID-19, LDH, Acute respiratory failure, Biomarkers

## Abstract

**Objective:**

The dramatic spread of SARS-CoV-2 infections calls for reliable, inexpensive tools to quickly identify patients with a poor prognosis. In this study, acute respiratory distress syndrome (ARDS) was assessed within 72 h after admission of each of 153 consecutive, SARS-CoV-2 infected, adult patients to either of two hospitals in Tenerife, Spain, using suitable routine laboratory tests for lymphocyte counts, as well as ferritin, lactate dehydrogenase (LDH), and C-reactive protein levels. Results were correlated with the patients’ respiratory function, defined through their pulse oximetric saturation/fraction of inspired oxygen (SpO2/FiO2) ratio.

**Results:**

Within 72 h from admission, criteria matched ARDS (SpO2/FiO2 < 235) in 13.1% of cases. We found a significant, negative correlation between SpO2/FiO2 ratios and d-dimer, ferritin, and LDH levels (− 0.31, − 0.32, and − 0.41; p = 0.004, 0.004, and < 0.0001, respectively). In patients with ARDS, the mean LDH was 373 U/L (CI_95%_: 300.6–445.3), but only 298 U/L (CI_95%_: 274.7–323.1) when they did not develop the syndrome (p = 0.015). None of the additionally evaluated biomarkers correlated with the SpO2/FiO2 ratios. Serum LDH levels in patients hospitalised for COVID-19 correlate with ARDS, as defined by their SpO2/FiO2 ratio, and might help to predict said complication.

## Introduction

The World Health Organization (WHO) has declared SARS-CoV-2 infection (COVID-19 disease) a pandemic [1]. Although current evidence suggests that most infections manifest mildly, up to 16% of cases may require hospital admission for developing severe pneumonia, acute respiratory distress syndrome (ARDS), sepsis, and even septic shock [2, 3]. An analysis of a cohort selected from 1099 COVID-19 patients throughout China showed that up to 15% of severe cases develop ARDS [2], which in turn becomes the main reason for their admission to an intensive care unit (ICU).

Given the unpredictable clinical course, multiple studies have focused on criteria that may correlate with a poor prognosis. Radiological findings point to COVID-19 patterns and extension as of prognostic value [4, 5]. In addition, serum biomarkers, such as leukocyte and lymphocyte counts, lactate dehydrogenase (LDH), d-dimer, troponin I, and ferritin levels seem to indicate the severity of the process and hence the need for ICU admission or may even predict mortality [6–10]. The pulse oximetric oxygen saturation/fraction of inspired oxygen (SpO2/FiO2) ratio is a simple measure, conventionally used in the context of ARDS [11]. Moreover, it seems to identify severely SARS-COV-2 infected patients who are at a high risk of death [12].

Hence, correlating biomarkers with complications, particularly ARDS, is of vital importance in patients who require hospital admission for COVID-19 disease. This study aimed to (1) evaluate the clinical and analytical characteristics of a cohort of patients, diagnosed for severe SARS-CoV-2 infection and therefore admitted to either of two hospitals in the island of Tenerife, Spain, from 1 March to 31 May, 2020 and (2) pinpoint biomarkers that correlate with ARDS occurrence within 72 h after admission of these patients to a regular ward.

## Main text

### Methods

#### Study design

This was a multicentre, retrospective, observational and descriptive, cross-sectional study including patients admitted with diagnosed, severe SARS-CoV-2 infection to either of two hospitals (Hospital Universitario Nuestra Señora de Candelaria and Hospital San Juan de Dios) in the island of Tenerife, Spain, from 1 March to 31 May, 2020.

#### Study population

The following inclusion criteria were applied: (1) patient age ≥ 18 years, (2) confirmed diagnosis of COVID-19. Diagnosis was obtained through SARS-CoV-2 real-time reverse polymerase chain reaction (RT-PCR) with samples from nasopharyngeal swabs. (3) first admission in one of the two participating hospitals. Patients were excluded (1) when the RT-PCR result was not positive despite symptoms and radiological findings compatible with COVID, (2) in case of active neoplasia, (3) when the patient required another, subsequent admission, (4) when they were directly admitted from the emergency room to the ICU due to disease severity.

Patients were treated at their attending doctor’s discretion, according to local protocols and clinical judgement. Some patients have been included in some other analysis that we sent for publication.

#### Ethical approval

The study was approved by the ethics committee of the University Hospital Nuestra Señora de Candelaria CHUNSC_2020_45.

#### Variables

The following variables were collected at hospital admission: (1) the demographic data age, sex, and smoking habit (active smokers if they had smoked at least one cigarette in the last 6 months, former smokers if they had smoked in the past but were remaining abstinent for at least 6 months, or non-smokers if they had never smoked); (2) the comorbidities arterial hypertension (AHT), dyslipidaemia (DLP), type 2 diabetes mellitus (T2DM), chronic obstructive pulmonary disease (COPD), asthma, ischaemic heart disease, atrial fibrillation, previous neoplasia, chronic kidney disease, and liver disease (the Charlson comorbidity index was calculated individually) [13]; (3) symptoms and findings from physical examination; (4) time lag between symptom onset and hospital admission; (5) total number of leukocytes, lymphocytes, and platelets, d-dimers, LDH, creatinine, aspartate aminotransferase (AST), alanine aminotransferase (ALT), gamma-glutamyl transpeptidase (GGT), alkaline phosphatase (ALP), C-reactive protein (CRP), sodium (Na), and potassium (K); (6) severity of the disease at admission by calculating the CURB65 score (confusion, urea, respiratory rate, blood pressure, age; range 0–5) [14] and the SpO2/FiO2 ratio [11]. An SpO2/FiO2 ratio  < 235 was considered indicative for ARDS, which corresponds to a PaO2/FiO2 ratio (ratio of partial pressure of arterial oxygen in mmHg to the fraction of inspired oxygen)  < 200 (moderate to severe ARDS) [15].

During follow up, the following variables were collected: (1) the serum parameters total number of leukocytes, lymphocytes, and platelets as well as d-dimer, ferritin, and LDH levels at 72 h from admission; (2) gas exchange at 72 h through the SpO2/FiO2 ratio; (3) the time lag between admission and transfer to the ICU or death or between admission to a regular ward and hospital discharge.

Follow up was terminated when patients were transferred to the ICU, died, or were discharged from a regular ward.

### Statistical analysis

Continuous variables were summarised as means and 95% confidence intervals (CI_95%_). Because of their asymmetric distribution, biomarkers were described as medians and quartiles 1 and 3. Qualitative variables were expressed as percentages. The non-parametric Mann–Whitney U-test was applied to compare biomarker distribution in patients with and without ARDS. Pearson’s correlation was used to define associations between variables. Differences at p < 0.05 were considered significant. Analyses were carried out using SPSS v.21.

### Results

A total of 160 patients were admitted to a regular hospital ward during the study period. Finally 153 patients were included, as 7 (4.3%) had to be excluded due to active neoplasia. The population characteristics are given in Table 1. The mean age was 67.3 years (CI_95%_ 64.8–69.9), 55.5% were men with a Charlson comorbidity index of 1.87 (CI_95%_ 1.42–2.32). The most frequent comorbidities were AHT (51.6%), DLP (39.8%), and T2DM (24.8%). Cardiac comorbidities (acute myocardial infarction, atrial fibrillation, and heart failure) were detected in 26.8% of the patients.

**Table 1:** Baseline characteristics of the patient population at hospital admission

Age (years), mean (CI_95%_)	67.3 (64.8–69.9)
Sex	
Men, n (%)	85 (55.56)
Women, n (%)	68 (44.44)
Comorbidities	
Arterial hypertension, n (%)	79 (51.63)
Dyslipidaemia, n (%)	61 (39.87)
Type 2 diabetes mellitus, n (%)	38 (24.84)
Atrial fibrillation, n (%)	20 (13.07)
Ischaemic heart disease, n (%)	24 (15.69)
Heart failure, n (%)	13 (8.50)
Chronic kidney disease, n (%)	15 (9.80)
Cerebrovascular accident, n (%)	11 (7.19)
Active smoker, n (%)	7 (4.58)
Chronic obstructive pulmonary disease, n (%)	12 (7.84)
Asthma, n (%)	15 (9.80)
Chronic liver disease, n (%)	5 (3.27)
Charlson index (not age adjusted), mean (CI_95%_)	1.87 (1.42–2.32)
Severity	
CURB65, mean (CI_95%_)	1.29 (1.15–1.44)
Oximetric and analytic parameter	Mean (CI_95%_)
SpO2/FiO2	423.3 (412.8–433.8)
Creatine (mg/dL)	1.10 (0.99–1.20)
Aspartate aminotransferase (U/L)	52 (38–66)
Alanine aminotransferase (U/L)	62 (37–88)
Gamma-glutamyl transpeptidase(U/L)	76 (61–92)
Alkaline phosphatase (IU/L)	85 (76–94)
Sodium (mmol/L)	138 (137–139)
Potassium (mmol/L)	4 (3.9–4)
Leukocytes (× 10^6^/L)	7509 (6469–8550)
Platelets (× 10^6^/L)	207,650 (194,819–220,482)
Lymphocytes, total (× 10^6^/L)	1612 (786–2439)
d-dimers (ng/mL)	1535 (899–2170)
Lactate dehydrogenase (U/L)	311 (291–331)
C-reactive protein (mg/dL)	19.74 (3.87–35.60)
Clinical manifestations of SARS-CoV-2 infected patients	
Fever, n (%)	84 (54.9)
Dyspnoea, n (%)	95 (62.09)
Cough, n (%)	113 (73.85)
Expectoration, n (%)	27 (17.64)
Fatigue, n (%)	44 (28.75)
Diarrhoea, n (%)	29 (18.95)
Myalgia, n (%)	35 (22.87)
Ageusia, n (%)	2 (1.3)
Anosmia, n (%)	1 (0.65)

The most frequently observed clinical manifestations were cough (74%), dyspnoea (62%), and fever (55%). The full range of symptoms is summarised in Table 1. At hospital admission, fewer than 3% of the patients met the criteria for ARDS. As Table 1 shows, the mean SpO2/FiO2 ratio was 423.3 (CI_95%_ 412.8–433.8) and the mean CURB65 score 1.29 (CI_95%_ 1.15–1.44) at that point of time.

The mean follow-up period was 13.3 days (CI_95%_ 11.9–14.7). Six patients (3.9%) died and 19 (12.4%) were admitted to the ICU. The mean time from onset of symptoms to hospital admission was 6.2 days (CI_95%_ 5.6–7.0). As Table 2 shows, the mean time elapsed between regular ward admission and transfer to the ICU was 3.4 days (CI_95%_ 2.3–4.7).

**Table 2:** Follow up of SARS-CoV-2 infected patients

	Mean (CI_95%_)
Time from symptom onset to hospital admission (days)	6.2 (5.6–7)
Follow up since hospital admission (days)	13.3 (11.9–14.7)
Time from symptom onset to treatment (days)	8.2 (7.2–9.1)
Stay at a regular ward (days)	14.6 (13.2–16.1)
Time from hospital admission to exitus (days)	15.1 (4.8–25.5)
Time from hospital admission to transfer to intensive care unit (days)	3.4 (2.3–4.7)

Within 72 h after hospital admission, 13.1% of the patients fulfilled criteria for ARDS (SpO2/FiO2 < 235). We detected a significant, negative correlation between SpO2/FiO2 and d-dimer, serum ferritin, and LDH levels (− 0.31, − 0.32, and − 0.41 with p = 0.004, p = 0.004, and p < 0.0001, respectively). For the rest of the biomarkers, this correlation was not significant. LDH levels were differentially distributed and, moreover, differed significantly between patients with ARDS and those who did not develop the syndrome (p = 0.015). Mean LDH levels were 373 (CI_95%_ 300.6–445.3) in patients with ARDS vs 298 (CI_95%_ 274.79–323.17) in those without ARDS. Correlations between ARDS and the other biomarkers were not significant (Table 3).

**Table 3:** Correlation between serum biomarkers and ARDS

	ARDS		No ARDS		p-value
	Mean (CI_95%_)	Median (Q1–Q3)	Mean (CI_95%_)	Median (Q1–Q3)	
Lymphocytes (× 10^6^/L)	1106 (805–1407)	960 (745–1370)	1766 (715–2817)	1175 (845–1550)	0.374
Platelets (× 10^6^/L)	278,417 (212,964–343,869)	284,000 (185,500–344,500)	243,049 (224,641–261,457)	230,500 (168,000–299,000)	0.246
d-dimers (ng/mL)	7456 (− 5877 to 20,789)	740 (498–4606)	875.53 (671–1079)	616 (385–1101)	0.371
Ferritin (ng/mL)	903 (296–1509)	695 (518–1413)	651 (499–803)	424 (209–909)	0.199
Lactate dehydrogenase (U/L)	373 (300.6–445.3)	370 (286–465)	298 (274.7–323.1)	284.5 (209.5–335.5)	0.015
C-reactive protein (mg/dL)	8.6 (3.2–13.9)	3.3 (0–13.4)	6.1 (4.9–7.4)	3.8 (0.6–8.7)	0.89

ARDS: acute respiratory distress syndrome

### Discussion

Although population-based studies on COVID-19 have identified features that characterise an unfavourable disease course, the clinical progression of distinct, virus-infected patients is highly variable. Hence, identifying biological markers that predict individual risk is vital. Age, the presence of comorbidities (e.g., AHT and T2DM), lymphopenia, increased serum inflammatory biomarkers, as well as elevated AST and LDH levels have been correlated with ARDS in patients with COVID-19 [16–18]. In line with other studies [2, 19, 20], our patients, hospitalised for COVID-19, were predominantly male, over 65 years of age, non-smokers, and their most frequent comorbidities were AHT, DLP, and T2DM. Furthermore, elevated LDH levels in the course of 72 h following admission correlated with ARDS, as defined by the SpO2/FiO2 rates.

Although the characteristics of our local patient group with severe SARS-COV-2 infection resembled those described in a recent, national study [20], we observed a higher prevalence of T2DM. Even though there is not much difference between the T2DM prevalence in the Canary archipelago and the Iberian Peninsula, patients on the islands exhibit more severe forms of the disease [21]. In general, diabetics are more susceptible to a wide range of infections [22–27], being reasonable to think that these patients are more susceptible to suffer SARS-CoV-2 infections and may suffer a poor disease course involving hospital admission [28, 29]. In addition, prevalence of heart disease was quite high (26.8%) in our patients. A recent meta-analysis revealed an association between the occurrence of cardiovascular disease and a worse clinical evolution profile in COVID-19 (OR: 3.88; CI_95%_ 2.30–6.54) [30–32]. Decompensation in patients in our archipelago through such common, chronic pathologies could favour their need for COVID-19 related hospital admission.

The clinical course of SARS-COV-2 infection can be assigned to three stages: early infection, pulmonary phase, and hyper-inflammatory phase, each characterised by its own biochemical alterations [33]. The first stage occurs when the virus infiltrates the lung parenchyma, where SARS-CoV-2 affects ciliated bronchial epithelial cells by interacting with the angiotensin-converting enzyme 2. The pulmonary phase is characterised by establishing viral pneumonia, associated with localised inflammation in the lungs, lymphopenia and an increase in inflammatory biomarkers. At this point, most patients require hospitalisation. The third stage of COVID-19 disease is the most severe, with intense, systemic inflammation, a so-called cytokine storm, which progresses into ARDS [34]. At the latter stage, patients usually require transfer to the ICU, which will occur within 5 days after hospital admission [35, 36].

Laboratory parameters, such as leukocyte and lymphocyte counts, LDH, d-dimers, troponin I, or serum ferritin can provide information about the course of the infection and be related to the need of ICU admission or the risk of mortality [6–10]. Several studies have shown that SARS-CoV-2 infected patients with an unfavourable, clinical course have higher levels of interleukin-6 and ferritin than subjects with a milder course [9, 10]. Thus, both biomarkers were proposed for COVID-19 patient monitoring during hospitalisation [11]. As to d-dimers, ARDS is known to associate with a hyper-coagulable state [37]. d-dimer is an indirect marker of thrombin formation and, thus, reflects ongoing endovascular thrombotic processes [37]. Zhang et al. [38] described increased d-dimer levels to be associated with severe forms of COVID-19 [39]. Similarly, in a work by Han et al. [40], d-dimer levels in COVID-19 patients increased with disease severity.

In our study, rising LDH levels within 72 h from hospital admission correlated with the occurrence of ARDS. These data agrees with a study published by Poggiali et al., which describes a correlation of serum LDH and CRP concentrations, using the PaO2/FiO2 ratio as a marker of ARDS [18]. In that latter work, the ROC curve showed a sensitivity of 75% and a specificity of 70% in identifying ARDS at the LDH cut-off value of 450 U/L; the area under the curve was 0.76 (p < 0.0001). The enzyme LDH is involved in energy generation and its concentrations are higher in organs such as heart, liver, lungs, and kidneys than in other tissues. LDH is a general indicator of tissue damage and is considered an inflammatory marker [40]. LDH serum concentrations increase during acute lung damage [41]. Nonetheless, we did not observe any correlation between LDH and the other evaluated biomarkers.

To our knowledge, this is the first study to evaluate the relationship between blood biomarkers in patients with COVID-19 and the SpO2/FiO2 ratio as a diagnostic criterion for ARDS. In contrast to the PaO2/FiO2 ratio, obtaining the SpO2/FiO2 ratio does not require invasive methods, a fact that reduces the exposure of health care personnel to patients, when assessing the data.

### Conclusions

Taken together, in patients hospitalised for COVID-19, elevated LDH levels correlate with the occurrence of ARDS as determined from the SpO2/FiO2 ratio. Taking this tool into account could help to early detect this clinical complication.

## Limitations

One of the limitations of this study is the relatively small sample size, which may have been the reason for not being able to establish a clear cut-off for serum LDH for use as a predictive marker. However, as our data are in line with Poggiali et al. [18], LDH levels > 450 U/L should alert to patients at high risk of developing ARDS [18, 42]. In addition, the retrospective nature of the study could have incurred an information bias due to obtaining the variables from the patient’s medical records, although current standardisation of diagnostic criteria minimises this possibility. Finally, we focused our analyses on cases with moderate to severe ARDS, so that the correlation between the selected biomarkers and mild ARDS (SpO2/FiO2 < 316 and > 232) [15] was not analysed.

## Data Availability

The study protocol and data collection forms are available from the corresponding author upon reasonable request. The datasets generated and analysed during the current study are not available to researchers outside of the co-investigators due to data protection laws.

## Abbreviations

AST: Aspartate aminotransferase
ALT: Alanine aminotransferase
ALP: Alkaline phosphatase
ARDS: Acute respiratory distress syndrome
AHT: Arterial hypertension
CI_95%_: 95% Confidence interval
COPD: Chronic obstructive pulmonary disease
CRP: C-reactive protein
DLP: Dyslipidaemia
GGT: Gamma-glutamyl transpeptidase
ICU: Intensive care unit
K: Potassium
LDH: Lactate dehydrogenase
Na: Sodium
PaO2/FiO2: Partial pressure of arterial oxygen in mmHg to the fraction of inspired oxygen ratio
RT-PCR: Real-time reverse polymerase chain reaction
SpO2/FiO2: Pulse oximetric oxygen saturation/fraction of inspired oxygen ratio
T2DM: Type 2 diabetes mellitus
WHO: World Health Organization
